# *Zymomonas mobilis* Biofilm Reactor for Ethanol Production Using Rice Straw Hydrolysate Under Continuous and Repeated Batch Processes

**DOI:** 10.3389/fmicb.2019.01777

**Published:** 2019-08-07

**Authors:** Tatsaporn Todhanakasem, O-lan Salangsing, Piyawit Koomphongse, Sanya Kaewket, Pattanop Kanokratana, Verawat Champreda

**Affiliations:** ^1^Department of Agro-Industry, Faculty of Biotechnology, Assumption University, Bangkok, Thailand; ^2^National Metal and Materials Technology Center (MTEC), Klong Luang, Thailand; ^3^National Center for Genetic Engineering and Biotechnology (BIOTEC), Klong Luang, Thailand

**Keywords:** *Zymomonas mobilis*, biofilm reactor, lignocellulosic hydrolysate, multistage continuous culture, repeated batch, ethanol production

## Abstract

Plastic composited corn silk was developed as a biotic/abiotic carrier for *Zymomonas mobilis* biofilm formation for the purpose of ethanol production. Furthermore, we explored the use of rice straw hydrolysate as substrate in both multistage continuous culture and repeated batch processes and compared the ethanol production efficiency by two strains of *Z. mobilis*. Biofilm formed by bacterial strains *Z. mobilis* ZM4 and TISTR551 were detected, and its proficiencies were compared under various conditions by scanning electron microscopy (SEM) and crystal violet assays. The greatest biofilm formed by both strains was found on day five after the inoculation. *Z. mobilis* strain ZM4 grown in repeated batch biofilm reactors produced higher yields of ethanol than TISTR551 grown under the same conditions, while TISTR551 produced higher yields of ethanol in the multistage continuous process. The yields were highly maintained, with no significant differences (*p* < 0.05) among the three consecutive repeated batches. These experiments highlight exciting uses for agricultural byproducts in the production of ethanol using *Z. mobilis* biofilm reactors.

## Introduction

Lignocellulosic materials are abundant renewable resources that mainly contain cellulose, hemicellulose and lignin, in which cellulose and hemicellulose are the main carbohydrate sources. Cellulose is a long chain of crystalline glucose molecules, while hemicellulose is mainly composed of a mixture of five- carbon sugars. This makes lignocellulosic material a potential carbon source that contributes significantly to various bioprocesses to produce many value-added products, including bioethanol and other commodities without competition for food demand (Isikgor and Becer, 2015). Rice is the largest food crop in the world and supplies 20% of the world’s nutrient energy. Therefore, bioethanol and other commodities could be produced from many agricultural waste products obtained from rice milling. However, lignocellulosic materials are not readily accessible to enzymatic hydrolysis, and processing them requires physical, chemical and enzymatic pretreatments. The pretreatment processes cause an extensive modification of the lignocellulosic structure, which allows enzymatic hydrolysis to release monosaccharides that can be further fermented by ethanologenic microbes (Sun and Cheng, 2002; Mosier et al., 2005). The pretreatment processes commonly lead to the formation of toxic byproducts, including sugar acids (cellobionic acid and gluconic acid), phenolic compounds, aliphatic acids (acetic acid, formic acid, and levulinic acid), and furan aldehydes [furfural and 5-hydroxymethylfurfural (HMF)] mixed with hexose and pentose sugars, which further develop the deleterious effects on enzymatic and microbial biocatalysis activities. Consequently, this reduces the production efficiency in many bioprocesses (Björklund et al., 2002; Serrano-Ruiz, 2015).

*Zymomonas mobilis* is an ethanologenic microbe with high ethanol yield and tolerance, osmotolerance, high fermentation productivity, broad pH range to ferment sugar (pH 3.5–7.5), and is generally regarded as safe (GRAS) (Panesar et al., 2006; Yang et al., 2016). *Z. mobilis* is only capable of growing on glucose, fructose and sucrose, however, it exhibits high ethanol production though the Entner–Doudoroff pathway using glucose as a substrate. *Z. mobilis* can produce ethanol yields of up to 97% of theoretical yield and ethanol concentrations of up to 12% (w/v) in glucose fermentations (Rogers et al., 1979). While toxic components in lignocellulosic hydrolysates have been found to inhibit the growth and enzymatic activity on the saccharification and ethanol production in many bacteria and yeasts (Franden et al., 2013; Zeng et al., 2014), *Z. mobilis* biofilms show enhanced tolerance to these toxic inhibitors (Li et al., 2006).

Biofilms produced by this strain exhibit higher ethanol production from lignocellulosic hydrolysates than cell suspensions and show a greater bacterial survival when exposed to toxic inhibitors, while also maintaining a higher metabolic activity (Todhanakasem et al., 2014, 2016). Biofilms are microbial cell layers that live in a self-produced matrix or hydrated extracellular polymeric substance (EPS) and attach to solid supports. Biofilms are self-regenerating systems with high cell density and stability, resistance to contamination and toxic substances, high productivity due to high active biomass, ease of manipulation in continuous processes and cell recycling, which together can lead to economic benefits (Cheng et al., 2010). Immobilization of cells in the form of biofilms or self- immobilization during fermentation in broth would be beneficial due to high bacterial populations, lower fermentation times, high productivity, high cell stability, feasibility of continuous and repeated processing, resistance to the high concentration of substrate and product, low cost of recovery and eventual minimization of the chance of contamination (Yu et al., 2007). Microbial biofilms have been used to convert various agricultural materials to value added products, including alcohol, organic acids, enzymes, amino acids, antibiotics, polysaccharides, exopolysaccharides and surfactants (Schmid et al., 2001; Straathof et al., 2002; Woodley, 2006; Pollard and Woodley, 2007). Cell densities can be expected to remain stable for a long period, and immobilized cells in the biofilms appear to be less affected by contamination (Muffler and Ulber, 2014). The application of biofilm reactors should minimize the complexity of the upstream process in preparation of lignocellulosic material, since removal of toxins prior to fermentation would no longer be required, thus minimizing production costs. However, biofilm reactors and processes need to be optimized to each specific organism prior to use in each industrial-scale production. In the past, immobilized *Z. mobilis* using Ca-alginate and PVA have been effectively used for ethanol production from lignocellulosic feedstock and were shown to produce up to 69.96% ethanol yield (Wirawan et al., 2012). This method relies on entrapment that encloses the catalyst behind membranes or within a gel structure and therefore creates diffusional resistance. While this helps prevent loss of cell viability, it also leads to loss of productivity. Biofilm reactors emerged as a new technology to overcome these problems (Vega et al., 1988). For example, continuous biofilm reactors of *Saccharomyces cerevisiae* outperformed suspension-culture reactors, with 15- to 100-fold higher ethanol productivities (gL^–1^ h^–1^) when glucose in rich medium was used as a substrate (Kunduru and Pometto, 1996a; Chen et al., 2013). Additionally, ethanol production by *Z. mobilis* biofilms was threefold higher than suspended cultures when glucose in rich medium was used as a substrate for fermentation under the batch process (Kunduru and Pometto, 1996b). Biofilm reactors with *Z. mobilis* have been developed into fluidized bed and packed bed reactors using different types of carriers to produce ethanol from starch or glucose-rich medium (Weuster-Botz, 1993; Weuster-Botz et al., 1993; Kunduru and Pometto, 1996b). There are many ways to operate biofilm reactors, including fluidized bed reactors, trickling filters, packed bed reactors, static volumetric flasks or submerged bed, stirred tank bioreactors, rotary disc reactors and membrane biofilm reactors. These reactors can then be operated in various ways, including batch, fed batch, repeated batch and continuous cultures that are appropriate to the productions (Kunduru and Pometto, 1996a; Ho et al., 1997a,b; Khiyami et al., 2006). Another attractive feature of biofilms is that bioprocesses can be manipulated using cheap raw materials, such as lignocellulosic materials, whey, molasses, industrial waste and glycerol waste from the biodiesel industry (Jun et al., 2010; Napoli et al., 2010; Todhanakasem et al., 2014). This is especially important given that raw material costs account for up to 70% of the final production cost. However, there have been no reports regarding applying biofilm reactors with lignocellulosic materials containing toxic inhibitors. Our previous studies show that shaking flask reactors using *Z. mobilis* biofilms and DEAE cellulose to produce ethanol from lignocellulosic material containing toxic inhibitors can result in ethanol production efficiency of up to 60% theoretical yield (Todhanakasem et al., 2015).

The surfaces and particles supplied in vessels for biofilm formation are crucial, as they should favor the adhesion of microorganisms, be inexpensive and widely available. In addition, the roughness, dimensions and porosity of the supports are also of great concern. The supports can be either inorganic or organic compounds. In the lab-scale production of ethanol from *S. cerevisiae*, wild sugarcane and silk cocoons have been used as biotic supporters for cell immobilization to operate under batch and repeated batch processes. However, the use of pure agricultural material as a carrier in the biofilm reactor may not be durable or recyclable for many rounds of production (Chandel et al., 2009; Rattanapan et al., 2011). Previously ethanol production was studied in *Z. mobilis* biofilms from various glucose-rich substrates in packed bed and fluidized reactors using different kinds of supports, such as anion exchange resin beads, macroporous glass beads and plastic composited supports (Kunduru and Pometto, 1996b). Inorganic material could be used as a carrier and would be more advantageous in terms of large specific surface area, narrow pre-distribution and good resistance against biodegradation (Galani et al., 1985).

Our earlier studies evaluated *Z. mobilis* biofilm reactors in ethanol production from lignocellulosic materials (Todhanakasem et al., 2014, 2015, 2016, 2018). This system not only increased ethanol productivity and yield, but also provided a cell recycling system and facilitated product recovery that further minimized production costs. Biofilm reactors can enhance bioconversion rates and yield from lignocellulosic biomass, and this activity can be maintained through cell recycling. The goal of the current study was to evaluate the use of plastic composited corn silk as a carrier for biofilm production and explore the use of rice straw hydrolysate as a substrate for ethanol production by *Z. mobilis* biofilm strains ZM4 and TISTR551 under continuous and repeated batch processes. The production efficiency was assessed by studying the ethanol yield (Y_P/S_).

## Materials and Methods

### Bacterial Strain and Cultivation

*Zymomonas mobilis* strains ZM4 (NRRL B14023) and TISTR 551 (Thailand Institute of Scientific and Technological Research, TISTR) were used for these studies. Prior to use as inoculums in any study, the cultures were grown in RMG glucose medium (10 g/L yeast extract, 2 g/L KH_2_PO_4_ and 2% w/v glucose, pH 5.8) at 30°C for approximately 24 h until the optical density at 600 nm (OD_600_) reached an absorbance of about 1.0.

### Pretreatment and Enzymatic Hydrolysis of Rice Straw

Rice straw was collected from Ladkrabang, Thailand. The materials were physically chopped using a cutting mill (Retsch ZM200, Retsch, Germany) and were then sieved to isolate particles sized 500 μm in diameter. Pretreatment was performed by the liquid hot water technique using a multi-reactor system (6 × 50 ml reactors) in a temperature-controlled jacket with a vertical shaking system. The temperature for the pretreatment was operated at 200°C for 7 min with substrate loading of 10% (w/v), and the initial pressure was 25 bars under nitrogen. The reactor was then quenched in a water bath. The pretreated rice straw was then separated by filtration and thoroughly washed with distilled water on a Buchner funnel until it reached pH 5.0–6.0. The sample was oven dried at 60°C prior to the process of enzymatic hydrolysis, which was performed by using a commercial enzyme mixture [10 FPU/g CTEC 2 (Novozyme, Denmark) supplemented with 330 IU/g OPTIMASH BG (CMCase) (Dupont, United States) and 120 IU/g OPTIMASH BG xylanase activity] (Dupont, United States) at 50°C for 72 h (Todhanakasem et al., 2018).

### Carrier Preparation

Corn silk was cut into small pieces (approximately 2–3 mm). The fine particles were removed by repeatedly suspending in water and discarding the unsettled particles. The delignification process was accomplished by incubating corn silk with potassium hydroxide (KOH) at a ratio of 10:1 (w/w) and then soaking in 0.01% acetic acid solution at 70°C for 1 h (Todhanakasem et al., 2016). The process was repeated 3–4 times. The delignified corn silk was separated using sheet cloth and oven dried at 70°C, then mixed with plastic without rubber (GP110 or general purpose plastic, Polimaxx) at a ratio of 1:4 (w/w). The mixture of plastic and delignified corn silk was further combined with 10% overnight-dried corn powder (w/w of the total mixture) as a binder. The plastic composite supports were prepared by adding the final mixture to the extruder hopper and proceeding through high temperature extrusion. The barrel temperatures were in the range of 170–220°C, and the spin speed was 40 rounds/min. The supports were then pelletized by cutting into 2 mm pieces (Kunduru and Pometto, 1996b). The specific surface area and porosity of the material was analyzed using the Brunauer Emmett and Teller (BET). A total of 100 g of carriers was oven dried at 60°C overnight and analyzed using the BET analyzer (Quantachrome/Autosorb-1 MP, United States), with conditions set at 100°C outgas temperature for 24 h, 2 P/Po Toler, an equilibration time of 3, a bath time of 77.35 and an analysis time of 154 min.

### Biofilm Development on Plastic Composite Corn Silk

Biofilm development on the plastic composited corn silk carriers (GP110 corn silk) was quantitatively and qualitatively analyzed. ZM4 and TISTR551 biofilms were allowed to develop on the supports by adding 10 g carrier to 20 ml of biofilm medium [20 g glucose, 5 g yeast extract, 5 g (NH_4_)_2_SO_4_, 0.6 KH_2_PO_4_, 0.4 g Na_2_HPO_4_⋅12H_2_O, 0.2 g MgSO_4_⋅7H_2_O and 0.01 g CaCl_2_ per liter], inoculating with 10% inoculum and allowing them to incubate at 30°C for 24 h under static conditions. The biofilm medium was replaced every day for 7 days. For crystal violet staining, 1 g of carriers with bacterial attachments was collected on days 0, 3, 5, and 7 to quantitatively analyze the rate of *Z. mobilis* attachment. Samples were stained with 1% (w/v) crystal violet for 1 min, washed with water for 5 min to remove excess dye and oven dried at 70°C overnight. The samples were then de-stained with 2 ml of 95% absolute ethanol for 5 min. A control without bacterial inoculation was set up for comparison. The OD_600_ absorbance was monitored and the intensity of crystal violet stain was correlated to the quantity of the bacterial attachment on the carriers after subtracting the intensity of the control. The final weight of attached cells or biofilm was determined by culturing *Z. mobilis* with 10 g of carrier and 20 ml of biofilm medium, inoculating with 10% inoculum and allowing to grow for 5 days. The final weight was obtained by discarding the liquid and oven-drying the supports at 70°C overnight. The bacterial attachment weight was calculated by subtracting the mass of the dried carrier from the dried mass of attached cells adsorbed on the plastic composited corn silk (w/w) and is referred to as cell retention of the carrier.

The qualitative analysis of *Z. mobilis* biofilm development on GP110 corn silk carrier was performed on days 0, 3, 5, and 7 using a scanning electron microscope (SEM). A one-gram sample was oven-dried at 70°C, then freeze-dried at −30°C for 6 h, 5°C for 8 h, 15°C for 7 h, and 30°C for 5 h. The sample was coated with gold prior to SEM analysis with a 0.8 kV sputtering voltage, 20 mA current, 5 cm working distance and 0.1 mbar vacuum for 30 s. SEM (Hitachi SU6600, United States) was used with a magnification of 5000× to visualize the samples.

### Identification of the Optimal Dilution Rate for Biofilm Reactors

Before operating the continuous process, the ideal dilution rate was determined based on the maximum specific growth rate of *Z. mobilis* biofilms. *Z. mobilis* was inoculated in 50 ml biofilm medium in a flask with a 10-fold dilution at pH 5.8 in the presence of 5% GP110 plastic composite corn silk. The biofilm was allowed to grow at 30°C for 5 days, replacing the biofilm medium every day. After 5 days, 1 L formulated medium at pH 5.8 containing the same amount of toxic inhibitors as the rice straw hydrolysate was added to the biofilm culture (furfural 4.58 mg/L, acetic acid 770.11 mg/L, syringaldehyde 7.3 mg/L, vanillin 2.27 mg/L, levulinic acid 2290.3 mg/L, formic acid 3611.89 mg/L, 5-HMF 1.967 mg/L, glucose 40 g/L, yeast extract 6.25 g/L, peptone 6.25 g/L). This formulation of toxic inhibitor media was based on our previous studies (Todhanakasem et al., 2018). Samples were collected every day to monitor glucose utilization using a glucose LiquiColor kit (Human, Germany). The reduction of glucose was used to indirectly evaluate the metabolic rate of biofilms to determine the maximum specific growth rate (μ_m_) and the doubling time (*t*_d_) (Rossell et al., 2002).

### Fermentation of Rice Straw Hydrolysate Into Ethanol by *Z. mobilis* in Biofilm Reactors Under the Multistage Continuous Process

**FIGURE 1:**
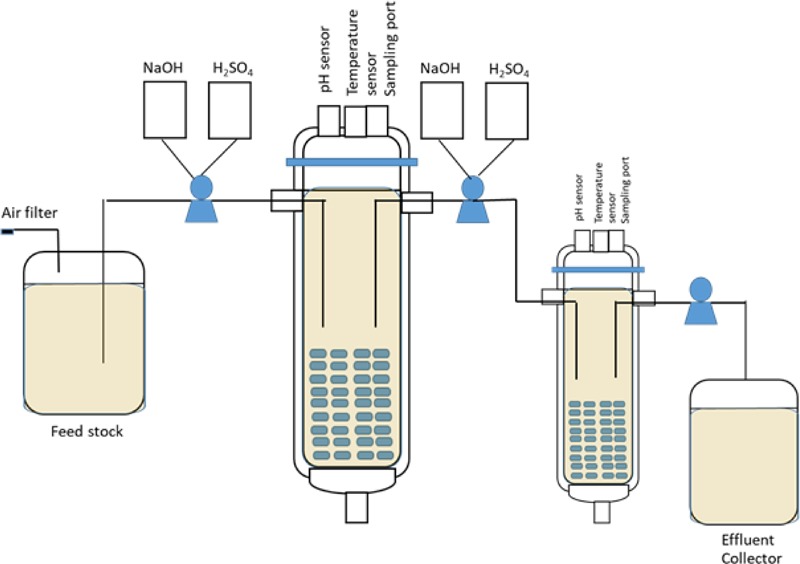
Schematic diagram of the experimental setup of *Z. mobilis* biofilm reactor under the multistage continuous process.

The laboratory-scale packed bed biofilm reactor with a multistage continuous process was designed to ferment rice straw. *Z. mobilis* ZM4 and TISTR 551 were analyzed for their ethanol production efficiency in multi-chamber biofilm reactors under continuous processes with an optimal dilution rate (*D*_opt_). The multistage continuous culture system reactor consists of cylindrical bulbs filled with GP110 plastic composite silk as a biofilm supporter that is approximately 5% w/v (weight of the carrier per the volume) of the 1 L working volume fermenter (vessel 1) and 500 ml working volume fermenter (vessel 2) (Figure 1). A total of 10% of the overnight cultures of ZM4 and TISTR551 were inoculated through the inoculum ports into the biofilm reactor, which was filled with polystyrene composite corn silk and biofilm medium at pH 5.8. The pH and temperature were controlled at 5.8 and 30°C and the reactors were run for 5 days with daily changes of biofilm medium. After 5 days, the biofilm medium was replaced with rice straw hydrolysate, which was used as a medium for ethanol production. The culture was processed under batch conditions until the glucose concentration was reduced to one half of its initial concentration, at which point the feeding was started. Glucose and ethanol were monitored every 24 h. The ethanol production efficiencies (Y_P/S_) of ZM4 and TISTR551 using rice straw hydrolysate under the multistage continuous process were monitored and calculated. The experiment was performed in triplicate and data were calculated based on mean values.

The concentration of ethanol was analyzed using gas chromatography (GC, HP Innowax Agilent 6890N) using an Innowax column (29.8 m × 0.25 mm × 0.25 μm) with a flame ionization detector (FID). The column temperature was 150°C, with a program run time of 5.5 min with ethanol retention time of about 1.9 min, a nitrogen carrier gas (16 kPa), an injector temperature of 175°C, detector temperature of 250°C, flow rate of 40 ml/min, split ratio of 1:50, a velocity of H_2_ flow of 60 ml/min and a sample quantity of 1 μl. The supernatant was filtered through 0.22 μm cellulose acetate filters prior to the GC analysis. Ethanol standard solutions were prepared at 0.1, 0.3, and 1.0% (v/v) using 95% absolute ethanol (Todhanakasem et al., 2018). The experiment was performed in triplicate.

### Fermentation of Rice Straw Hydrolysate Into Ethanol by *Z. mobilis* Biofilm Reactors Under the Repeated Batch Process

The laboratory-scale packed bed biofilm reactor was operated under the repeated batch process. The biofilm reactor was inoculated with *Z. mobilis* in biofilm medium at pH 5.8 at a 10-fold dilution in the presence of 5% GP110 plastic composite corn silk in 1 L working volume. The biofilm was allowed to grow at 30°C for 5 days, with replacement of the biofilm medium occurring each day. After 5 days, the biofilm medium was replaced with 1 L of rice straw hydrolysate and was fermented for another 3 days. The pH and temperature were controlled at 5.8 and 30°C. The repeated batch fermentations were first carried out under the batch mode. At the end of each batch, the fermented broth was decanted and the carrier with immobilized cells was retained in the bioreactor. Later, the same amount of the rice straw hydrolysate was immediately replaced to start the next cycle. A total of three consecutive batches under the repeated batch operation were processed and the ethanol yields (Y_P/S_) were monitored among the three batches. The experiment was performed in triplicate and the data was compared by statistical analysis using ANOVA with Duncan’s multiple range test. The null hypothesis was accepted or rejected with 95% confidence interval (*p* < 0.05).

## Results

### Analysis of Carriers and *Z. mobilis* Biofilm Development on the Carriers

**FIGURE 2:**
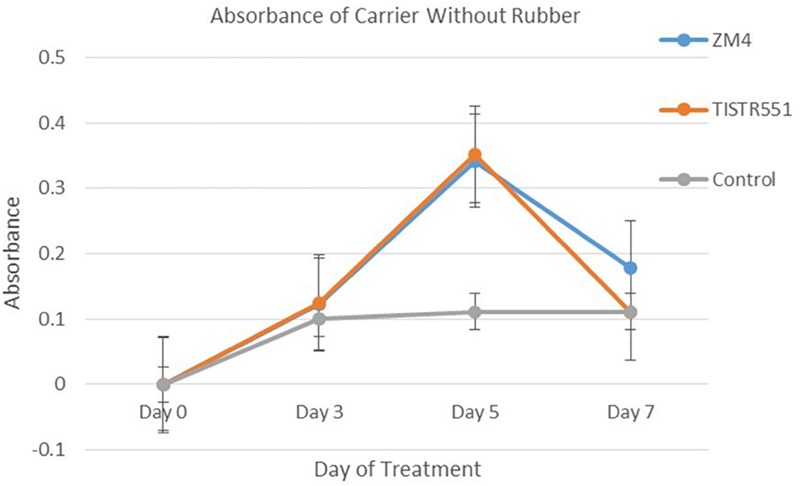
*Zymomonas mobilis* ZM4 and TISTR551 biofilm development or bacterial attachment on GP110 plastic composited corn silk on days 0, 3, 5, and 7, based on the 1% w/v crystal violet staining assay were compare with the control. The experiment was performed in triplicate, and the average and SD were plotted among these experiments.

**FIGURE 3:**
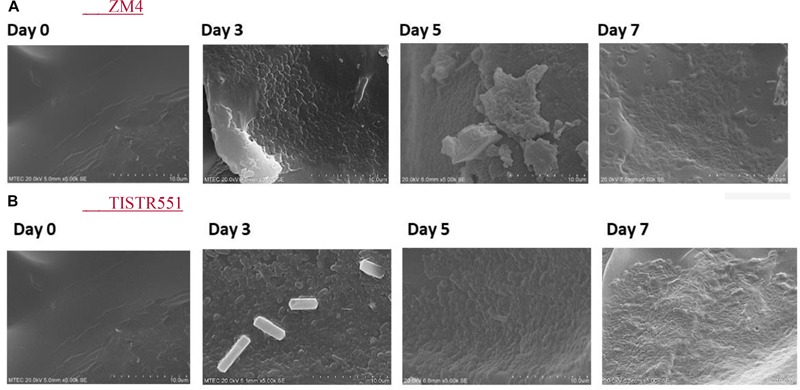
*Zymomonas mobilis* ZM4 **(A)** and TISTR551 **(B)** attachments on GP110 plastic composited corn silk on days 0, 3, 5, and 7 monitored by SEM with 5000× magnification.

*Zymomonas mobilis* ZM4 and TISTR551 were cultivated in a biofilm medium to allow development of biofilms on plastic composited corn silk carriers (GP110 plastic composited corn silk) with the multipoint BET 2.0 m^2^/g, total pore volume 0 cc/g and average pore diameter 39.1 Å (Table 1). In BET analysis, total pore volume and average pore diameter are used to determine the overall specific external and internal area, volume and porosity of the solid supports. The presence and abundance of biofilms formed by strains ZM4 and TISTR 551 were elucidated by crystal violet staining and scanning electron microscopy (SEM). Gram staining was performed on supports after the cultures were inoculated into biofilm medium and allowed to incubate for 24 h under static conditions. Carriers with the bacterial biofilms were quantitatively analyzed via the crystal violet assay and compared with un-inoculated supports on day 0, 3, 5, and 7 (Figure 2). The results obtained in the crystal violet biofilm staining assay were in agreement with the results obtained with SEM analysis (Figure 3). There was no bacterial attachment on day 0 under the SEM observation, which was correlated to the crystal violet staining where the absorbance at on OD of 600 was 0 right after the inoculation time. ZM4 and TISTR551 formed dense homogeneous structures of cell attachments distributed along the entire surface area on day 3 in the initial stage of biofilm development, and this correlated with an increase in the absorbance level of crystal violet. ZM4 and TISTR 551 showed significantly different morphological patterns of biofilms on plastic composited corn silk carrier under SEM analysis on day 5. In addition to the differences in pattern, the overall abundance of biofilm was also greater compared to day 3, and this was quantitated in the crystal violet staining assay. On day 5, ZM4 possessed a mature heterogeneous biofilm morphology while TISTR551 exhibited a homogeneous biofilm morphology. Both strains were embedded under extracellular polymeric substances (EPS). These two strains exhibited highly developed biofilms which then detached on day 7. The weights of ZM4 and TISTR551 cell retentions were 5.2 ± 0.6 and 6.3 ± 1.1 g, respectively, on day 5 (Table 1). The BET surface area, pore volume, and average pore diameter of plastic composited corn silk were low, but the biomass loading rate and biofilm formation were high by day 5, as shown by qualitative and quantitative assays, indicating the carriers provided sufficient interstitial spaces for cell entrapment or the biofilm formation. This suggests the chemical composition of supports, rather than the physical properties, of the supporting material could play a significant role in bacterial attachment.

**TABLE 1:** Analysis of the surface area of a carrier using Brunauer–Emmett–Telle analysis and the weight of ZM4 and TISTR551 cell retention on GP110 plastic composited corn silk.

**Sample name**	**Multipoint BET (m^2^/g)**	**Total pore volume (cc/g)**	**Average pore diameter (Å)**	**Weight of ZM4 cell retention (g)**	**Weight of TISTR551 cell retention (g)**
GP110 plastic composited corn silk	2.0	0	39.1	5.2 ± 0.6	6.3 ± 1.1

*The cell retentions were evaluated on day 5.*

### Fermentation Under the Multistage Continuous Process

**FIGURE 4:**
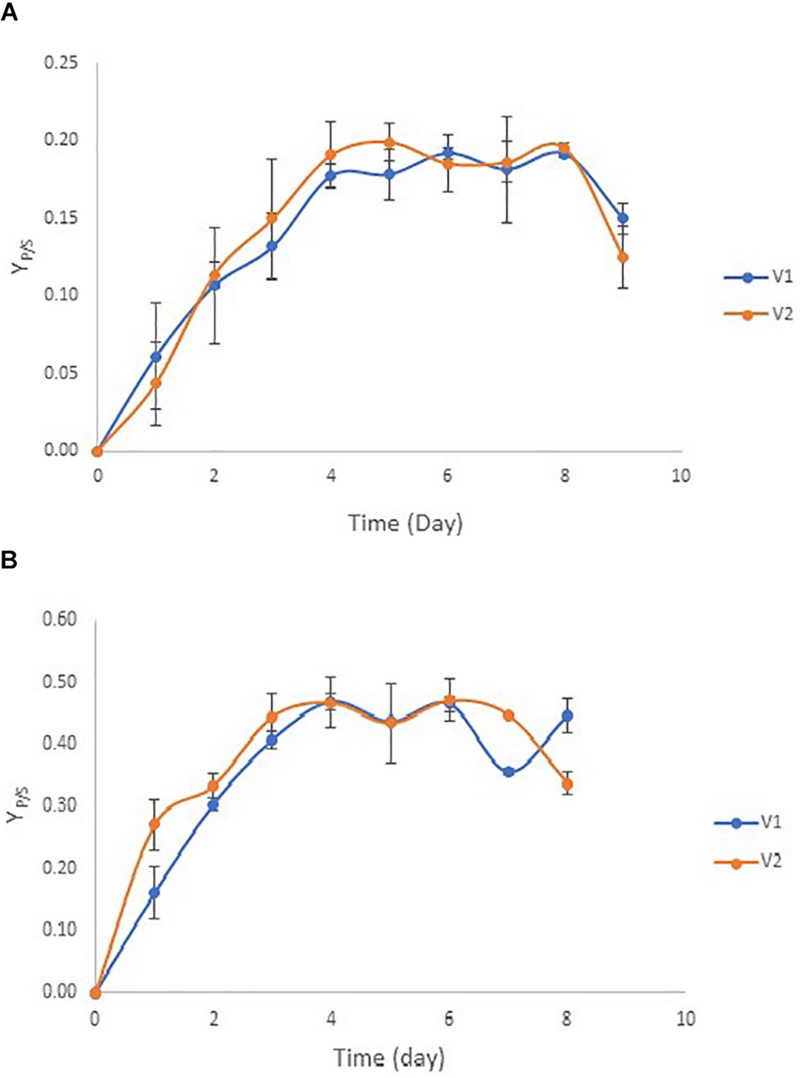
Ethanol yield (Y_P/S_) by biofilms of *Z. mobilis* strains ZM4 **(A)** and TISTR 551 **(B)** in multi-chamber biofilm reactors (V1 and V2) at the optimum dilution rate using rice straw hydrolysate as a substrate.

Chamber flow rates were controlled at 50% of the actual flow rate in vessel 1 (V1) and at full actual flow rate in vessel 2 (V2). The actual flow rates were calculated based on the maximum specific growth rates of ZM4 and TISTR551 biofilms, thus creating the optimal dilution rate. It was difficult to maintain a high dilution rate (over 50% of the actual flow rate) in V1 or to complete the consumption of glucose in just a single vessel when rice straw was utilized as a substrate because of the large toxic composition of the medium. These toxins reduced the overall glucose consumption rate and further reduced the ethanol yield (data not shown). Therefore, the biofilm reactors were designed as multistage continuous processes with the second bioreactor at half the volume of the first bioreactor to provide a doubling in dilution rate (*D*) in V2. This method ensured complete glucose utilization in rice straw hydrolysate (Figure 1). The continuous flow began on day 4 for *Z. mobilis* ZM4 biofilms with a flow rate of 37.8 ml/h and dilution rates of 37.8 h^–1^ in vessel 1 (V1) and 75.6 h^–1^ in vessel 2 (V2). Meanwhile, the flow started on day 2 for *Z. mobilis* TISTR551 biofilms with a flow rate of 13.5 ml/h and dilution rates of 13.5 h^–1^ for vessel 1 (V1) and 27 h^–1^ for vessel 2 (V2) (Figure 4 and Table 2). Prior to initiating flow, the ethanol yield (Y_P/S_) for *Z. mobilis* biofilm reactors was low. Flow was initiated when the remaining glucose concentration was reduced to one half of its initial concentration in the biofilm reactor. Cultures in the reactors were monitored for ethanol produced per amount of glucose consumed in order to determine the yield (Y_P/S_) and to evaluate the steady-state condition of the continuous process. Under the steady state condition, Y_P/S_ of ZM4 biofilms in vessel 1 (V1) and vessel 2 (V2) were 0.17–0.19 g/g (33.33–37.25% theoretical yield) and 0.18–0.19 g/g (35.29–37.25% theoretical yield), respectively, whereas TISTR551 biofilm reactors yielded (Y_P/S_) 0.35–0.47 g/g (68.63–92.16% theoretical yield) and 0.43–0.47 g/g (84.31–92.16% theoretical yield), respectively (Figure 4). Y_P/S_ of ZM4 biofilm reactors under the multistage continuous process could be maintained steadily for 5 days in both vessels, with the yield dropping approximately 22–36% on day 6 while the yield of TISTR551 biofilm reactor dropped approximately 33% only in V2.

**TABLE 2:** Summary of kinetics studies and ideal flow rate (continuous operation) of *Z. mobilis* ZM4 and TISTR551 biofilms.

**Biofilm (strain)**	**Doubling time (*t*_d_, hour)**	**Maximum specific growth rate (μ_m_, h^–1^)**	**Flow rate (F, ml h^–1^)**
ZM4	9.2 ± 0.1	0.08 ± 0.0	75.6 ± 10
TISTR551	25.7 ± 0.3	0.03 ± 0.0	26.9 ± 0.9

### Fermentation Under the Repeated Batch Process

**FIGURE 5:**
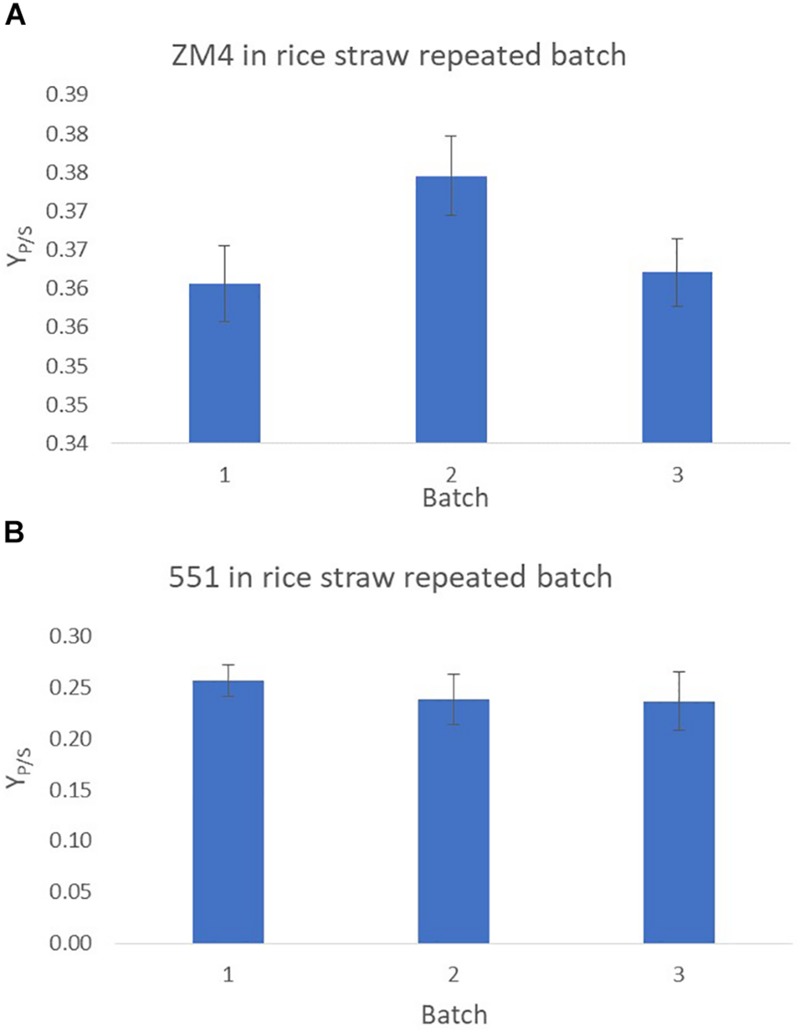
Repeated batch fermentation of biofilms for *Z. mobilis* strains ZM4 **(A)** and TISTR 551 **(B)** in rice straw hydrolysate. This was processed in a total of three consecutive batches. The experiments were performed in triplicate and the data were statistically analyzed using ANOVA.

The fermentation efficiency of *Z. mobilis* strains ZM4 and TISTR551 immobilized on plastic composite corn silk was evaluated under repeated batch process using rice straw hydrolysate as a substrate. The laboratory scale repeated batch was operated in the packed bed biofilm reactor of 1 L working volume. Ethanol yields (Y_P/S_) were calculated at the end of each fermentation for a total of 3 cycles of consecutive repeated batches (with 3 days in each cycle), with sterile fermentation medium replaced in each batch (Figure 5). Ethanol yields were calculated at the end of day 3 for each batch and were at 0.36–0.38 g/g (70.58–74.51% theoretical yield) for *Z. mobilis* ZM4 and at 0.24–0.26 g/g (47.06–50.98% theoretical yield) for TISTR551, respectively. There were no significant differences with *p* < 0.05 using ANOVA between the three consecutive batches (Figure 5). Ethanol yields remained stable in all three fermentation cycles, with a small reduction in the ethanol yield in the third cycle of the repeated batch.

## Discussion

Fermentation processes can be classified into two categories according to the morphological state of the microbial biomass. The first category is composed of fermentation processes promoting the growth of the microbial biomass in a planktonic state (suspended cells) in a liquid phase. These processes are mainly related to stirred tank reactors (STRs) and are widely used at the industrial scale for the production of biomass and metabolites. The second category of fermentation processes involves the growth of the microbial biomass as biofilms (sessile state) for various bioprocesses (Claessen et al., 2014). Until now, biofilm reactors have been commercially used in the treatment of wastewater, bioremediation and off-gas. Biofilms also have the potential for the sustainable production of bio-based materials through bioprocesses, as they are self-immobilized, highly resistant to toxic inhibitors, rapid fermenters, exhibit long-term activity, high cell concentration or density, are able to cell recycle and simplify downstream processes. These characteristics will further reduce overall production costs. For the past few years, biofilms have been considered effective biocatalysts and environmentally friendly tools for the industrial production of chemicals such as bioethanol, butanol, hydrogen, microbial fuel cells, enzymes, amino acids, antibiotics, polysaccharides, exopolysaccharides, surfactants, proteins and secondary metabolites (Schmid et al., 2001; Straathof et al., 2002; Pollard and Woodley, 2007).

In this study, we worked to design a *Z. mobilis* biofilm reactor suitable for process scale-up that would maximize productivity, increase long-term continuous productivity, reduce labor costs once steady state is reached, minimize waste production and energy consumption and simplify operation of the downstream processes, with the idea that the process should be able to operate continuously. *Z. mobilis* ZM4 and TISTR551 were cultivated in a biofilm medium to allow development of biofilms on plastic composited corn silk carriers. Biofilm development was analyzed by using crystal violet staining and scanning electron microscopy (SEM). Both strains exhibited highly developed biofilms on day 5, which then detached by day 7. The morphological structures of the biofilms that formed on the carriers were significantly different for the two bacterial strains. In both cases, the plastic composite corn silk provided the best substrate for cell attachment. The plastic composite agricultural materials provided nutritional benefit to the bacteria as the support broke down as well as a surface for cell attachment which promoted biofilm formation. This indicates that lignocellulosic materials which contain cellulose, hemicellulose and lignin from corn silk could provide additional nutrients for biofilm development (Kunduru and Pometto, 1996b; Todhanakasem et al., 2016). Our result indicated that cell immobilization, and therefore cell concentration, can increase up to day 5. *Z. mobilis* biofilms produce extracellular polymeric substances (EPS) that provide a diffusion barrier against toxic compounds that could harm the cells, thus resulting in increased cell densities and increased fermentation efficiency (Todhanakasem et al., 2014). Agricultural materials in carriers have been found to induce bacterial attachment by serving as a substrate source material (Kunduru and Pometto, 1996b; Todhanakasem et al., 2016). Plastic composited corn silk supports could stimulate cell attachment or biofilm development, as corn silk has been speculated to induce bacterial attachment better than plastic material (Kunduru and Pometto, 1996b; Todhanakasem et al., 2016). In our study, we suggest that surface area, porosity of the support and the presence of corn silk helped initiate biofilm formation, which then further increased ethanol production as a result of higher cell density. The cell detachment phenomenon may help to control cell overgrowth in the interstitial spaces, which could then minimize cell blockage which can be a problem for long term operation in packed bed bioreactors.

Continuous ethanol fermentations were performed in triplicate using the biofilm reactors of *Z. mobilis* ZM4 and TISTR551 in packed bed reactors operated under a multistage continuous process with rice straw hydrolysate. The reactor consists of a cylindrical bulb filled with GP110 plastic composited corn silk as a biofilm support, which was approximately 5% of the 1 L working volume (vessel 1 or V1) and 500 ml working volume (vessel 2 or V2). The flow rates were operated based on the calculation of the optimal dilution rate using the maximum specific growth rates for ZM4 and TISTR551 biofilms when kinetics were analyzed in the formulated rice straw medium. This model medium contained toxic inhibitors at levels found in rice straw hydrolysate and which include furfural, acetic acid, syringaldehyde, vanillin, levulinic acid, formic acid and 5-HMF. These inhibitory compounds have been reported to synergistically negatively affect the growth rate of microorganisms by disturbing cell membrane function, inhibiting essential enzymes, negatively interacting with DNA and RNA and specifically modulating the Entner Doudoroff pathway (Banerjee et al., 1981; Delgenes et al., 1996; He et al., 2012; Franden et al., 2013). In our study, flow rates were set to allow complete consumption of glucose and to increase the efficiency of ethanol production from lignocellulosic hydrolysate containing toxic inhibitors. Complete consumption of glucose is of great importance for industries in order to minimize substrate waste and maximize ethanol concentration and yield. The flow in the continuous process was begun when the glucose concentration dropped to that which is seen in the middle of the exponential growth phase in biofilm mode where glucose concentration was reduced to approximately one half of its initial concentration. Two biofilm reactors were designed with different volumes. The first reactor was twice the volume of the second reactor, and thus had a different *D* value. Industrial fermentation designs often use one fermenter that is twice the volume of a second fermenter in order to maximize the bacterial cell growth early in the multistage system (Bayrock and Ingledew, 2001). We found that continuous fermentation in a series of reactors that progressively increase dilution rates can enhance the efficiency of ethanol production in terms of ethanol concentration and product yield. In this study, the TISTR551 biofilm reactor provided a higher ethanol yield than the ZM4 biofilm reactor and we saw that ethanol was more effectively produced from glucose in the later stages (up to 92% theoretical yield). In addition, TISTR551 biofilms adjusted within a couple of days to the rice straw hydrolysate, while ZM4 biofilms required 4 days prior to starting the flow. Ethanol yields were equally produced in all stages of continuous culture. The maximum glucose concentration remaining in V2 was approximately 17 g/L for the ZM4 biofilm reactor and 2 g/L for the TISTR551 reactor. The steady state conditions could be prolonged for 5 days in both the ZM4 and TISTR551 biofilm reactors. Unknown environmental conditions can arise in the biofilm system during continuous culture, in addition to biofilm detachment when biofilms are exposed to the stress from lignocellulosic hydrolysate (Todhanakasem et al., 2018). This detachment phenomena may consequently lead to inconsistency of ethanol production after day 6. TISTR551 was found to be more tolerant to toxic inhibitors from lignocellulosic hydrolysate than ZM4 in the biofilm growth mode, thus potentially leading to higher ethanol yields than ZM4 (Todhanakasem et al., 2018). We hypothesize that the heterogenous nature of the ZM4 biofilm on plastic composited corn silk exposes it to shear forces in the continuous flow bioreactor, thus leading to biofilm detachment and further reduction in ethanol yield. Therefore, continuous culture with TISTR551 biofilm could be the technique that increases ethanol production from lignocellulosic hydrolysate. Continuous culture fermentation provides advantages over batch fermentations in terms of maximum productivity, long-term continuous productivity, and reduced labor cost once steady state is reached, and these are all beneficial for industrial-scale production. Multistage continuous culture was applied in this work to facilitate the completion of glucose utilization in order to minimize wastage of the substrate from lignocellulosic material and maximize ethanol concentration and yield, which is especially useful when toxic compounds exist in the lignocellulosic substrate.

Biofilm reactors under the repeated batch process are beneficial in that inoculum can be reused during various fermentation cycles, there is good depletion of medium in the reactor at the end of cultivation, there is high cell concentration in the culture, with low time required for process operation, and low risk of process contamination. Therefore, the fermentation efficiency of *Z. mobilis* strains ZM4 and TISTR551 immobilized on plastic composite corn silk were also evaluated under repeated batch process using rice straw hydrolysate as a substrate. The repeated batch fermentations were first carried out in batch mode. At the end of each batch, the fermented broth was drained and only the carrier with immobilized cells was retained in the bioreactor. The repeated batch process was performed for three cycles. Ethanol yields (Y_P/S_) were calculated at the end of each fermentation to evaluate the stability of plastic composited corn silk as a supporting material for ethanol production from rice straw hydrolysate. The ethanol yields were highly maintained among the three consecutive batches and exhibited no significant differences (*p* < 0.05) even though there was a slight reduction in production in the last batch. Therefore, *Z. mobilis* biofilm reactor for the production of ethanol from lignocellulosic hydrolysate could be repeated three times without any loss of fermentation ability. In the repeated batch process, we found no lag time for ethanol production at the start of each batch, indicating that the immobilized cells were vigorously metabolizing throughout the experiment (Watanabe et al., 2012). *Z. mobilis* ZM4 biofilm reactors produced a higher ethanol yield than the TISTR551 biofilm reactors under the repeated batch process. Additionally, the ethanol yield of ZM4 biofilm reactors in the repeated batch was superior to that of the continuous culture, possibly because the heterogeneous biofilm structure of this strain is more stable in static conditions. However, there was a repeated batch fermentation of formulated lignocellulosic hydrolysate to produce ethanol by metabolically engineered *S. cerevisiae* suspended culture where the process could be repeated five times without any loss of fermentation ability (Sanda et al., 2011). To our knowledge this is the first report of repeated fermentation of lignocellulosic hydrolysate to produce ethanol by *Z. mobilis* biofilm reactor. Repeated batch fermentation with the biofilm reactor is considered to be a promising method for cost-effective ethanol production due to the reduction in time and cost associated with inoculum preparation. Further studies using eight consecutive batches will be required to validate this method for industrial purposes, but this first study indicates there is merit in exploring plastic composited corn silk as a supporting material for ethanol production from rice straw hydrolysate.

In conclusion, biofilm reactors for *Z. mobilis* could be potentially used in bioprocesses, and we found that use of plastic composite support reactors can lead to enhanced ethanol production by stimulating cell attachment and biofilm formation. Multistage continuous technologies are insufficient in the published work and are needed to allow further exploration in this area, especially in terms of use of lignocellulosic materials. These results indicate that a substantial gain in ethanol production can be achieved with biofilm bioreactors. As a natural form of cell immobilization, biofilm allows for a potentially short startup period that exhibits high cell density, stability, allows for continuous operation, and gives high productivity while maintaining high metabolic activity and cell viability with easy downstream processing. For these specific reasons, biofilm reactors are ideal for industrial scale production applications. However, biofilm kinetics must be further studied in order to scale up the process.

## Data Availability

The raw data supporting the conclusions of this manuscript will be made available by the authors, without undue reservation, to any qualified researcher.

## Author Contributions

TT wrote the manuscript, designed the work, implemented the laboratory works, solved the problem, and analyzed and interpreted the data. O-lS, PK, and SK implemented the laboratory works. PK assisted with the laboratory works. VC consulted on the project. All authors were responsible for the content and writing of this manuscript.

## Conflict of Interest Statement

The authors declare that the research was conducted in the absence of any commercial or financial relationships that could be construed as a potential conflict of interest.
